# Recruiting general practitioners and older patients with multimorbidity to randomized trials

**DOI:** 10.1093/fampra/cmad039

**Published:** 2023-04-04

**Authors:** Caroline McCarthy, Ivana Pericin, Susan M Smith, Frank Moriarty, Barbara Clyne

**Affiliations:** HRB Centre for Primary Care Research, Department of General Practice, RCSI University of Medicine and Health Sciences, Dublin 2, Ireland; School of Social Work and Social Policy, Trinity College Dublin, Dublin 2, Ireland; HRB Centre for Primary Care Research, Department of General Practice, RCSI University of Medicine and Health Sciences, Dublin 2, Ireland; Department of Public Health and Primary Care, Trinity College, Dublin 2, Ireland; HRB Centre for Primary Care Research, Department of General Practice, RCSI University of Medicine and Health Sciences, Dublin 2, Ireland; School of Pharmacy and Biomolecular Sciences, RCSI University of Medicine and Health Sciences, Dublin 2, Ireland; HRB Centre for Primary Care Research, Department of General Practice, RCSI University of Medicine and Health Sciences, Dublin 2, Ireland

**Keywords:** multimorbidity, polypharmacy, process evaluation, randomized controlled trial, research design

## Abstract

**Background:**

Older patients with multimorbidity are under-represented in experimental research.

**Objective:**

To explore the barriers and facilitators to general practitioner (GP) and older patient recruitment and retention in a cluster randomized controlled trial (RCT).

**Method:**

This descriptive study uses qualitative and quantitative data from a cluster RCT, designed to evaluate the effectiveness of a medicines optimization intervention. The SPPiRE cluster RCT enrolled 51 general practices and 404 patients aged ≥65 years and prescribed ≥15 medicines. Quantitative data were collected from all recruited practices and 32 additional practices who were enrolled, but unable to recruit sufficient participants. Qualitative data were collected from purposive samples of intervention GPs (18/26), patients (27/208), and researcher logs and analysed thematically using inductive coding.

**Results:**

Enrolment rates for practices and patients were 37% and 25%, respectively. Barriers to GP recruitment were lack of resources and to patient recruitment were difficulty understanding trial material and concern about medicines being taken away. GPs’ primary motivation was perceived importance of the research question, whereas patients’ primary motivation was trust in their GP. All general practices were retained. Thirty-five patients (8.6%) were lost to follow-up for primary outcomes, mainly because they had died and 45% did not return patient-reported outcome measures (PROMs).

**Conclusion:**

Patient retention for the primary outcome was high, as it was collected directly from patient records. Patient completion of PROM data was poor, reflecting difficulty in understanding trial material. Recruiting older patients with multimorbidity to clinical trials is possible but requires significant resource and planning.

**Trial registration:**

ISRCTN Registry ISRCTN12752680.

**Key messages**

- Older people with multimorbidity are under-represented in experimental research.
- Primary care is the ideal setting for including this population in RCTs.
- GPs participate in RCTs when they perceive the research to be important.
- Trust in their GP facilitates recruitment of older people with multimorbidity.
- Disease and treatment burden are barriers to retention in this population.
- The collection of patient-reported outcomes measures needs careful consideration.

## Introduction

As the population of older people living with multimorbidity and associated polypharmacy continues to rise^1^ the safe and equitable distribution of effective therapeutic interventions will become an increasingly pressing health policy priority.^2^ Multimorbidity and polypharmacy are associated with adverse outcomes including reduced quality of life, adverse drug reactions and unplanned hospital admissions.^3–6^ Clinicians describe difficulty with clinical decision-making for older patients with multimorbidity and polypharmacy,^7^ who are typically excluded from interventional studies that form the basis of clinical guidelines.^8^ It is important that older patients with multimorbidity are included in these studies, but randomized controlled trials (RCTs) often face difficulties with recruitment and retention, and this is especially the case for hard to reach groups such as older patients with complex multimorbidity.^9–11^

Barriers to patient recruitment in primary care may be categorized into 4 areas: study-related, patient-related, practitioner-related, and practice- or organizational-related.^12^ Patient-related reasons for non-participation in clinical research include restrictive eligibility criteria, inconvenience, fear of adverse effects, lack of interest, and language difficulties.^13–15^ Organizational- and study-related barriers impact on practitioner-related barriers which primarily relate to difficulty prioritizing research due to other competing demands.^12,14^ Qualitative studies from various settings have indicated general practitioners (GPs) are often willing and interested in taking part in research, particularly if the research question is considered relevant but that inadequate time, resources, and planning are barriers to participation.^16,17^ The Supporting Prescribing in Older Patients with Multimorbidity (SPPiRE) cluster RCT was designed to assess the effectiveness of a complex intervention in reducing polypharmacy and improving the quality of prescribing in patient with multimorbidity aged ≥65 years and prescribed ≥15 repeat medicines.^18^ The trial recruited its target sample size and the intervention had a small but significant effect in reducing the number of medicines but no effect on the overall quality of prescribing.^19^ This study was unique in that its target population were a group with very significant disease and treatment burden who are often excluded from clinical research. The aim of this study was to explore practice and patient recruitment and retention to the SPPiRE cluster RCT, including enrolment rates, characteristics of recruited practices and patients and both GP and patient perceptions of the barriers and facilitators of recruitment. A secondary aim was to identify recommendations for conducting future research with this population.

## Methods

### Study setting and context

Data for this study were collected in the SPPiRE cluster RCT and the mixed methods parallel process evaluation, described in detail in their published protocols and results papers^18–21^ (see Supplementary Fig. 1). Full ethical approval was granted by the Irish College of General Practitioners and all GPs and patients gave fully improved written consent. This current paper describes patient and GP recruitment and retention using both qualitative and quantitative data.

### Recruitment process

Practices throughout the Republic of Ireland were informed about the SPPiRE trial through a variety of sources. Eligible practices (those with at least 300 patients aged ≥65 years on their patient panel) who expressed an interest were formally invited to take part. Recruited practices identified eligible patients aged ≥65 years and prescribed ≥15 medicines by running a finder tool that was embedded in practice software. The target cluster size was reduced from 15 to 5 in early recruitment when it became apparent practices were unable to recruit to the original target. Study personnel had no access to patient data prior to the patient consent process so recruitment packs were posted to practices with the required number of patient invitations. Multiple batches of invitations were sent to larger practices to prevent over recruitment. Practices were sequentially randomized once participant recruitment and baseline data collection was complete.

### Study population

**Fig. 1.**
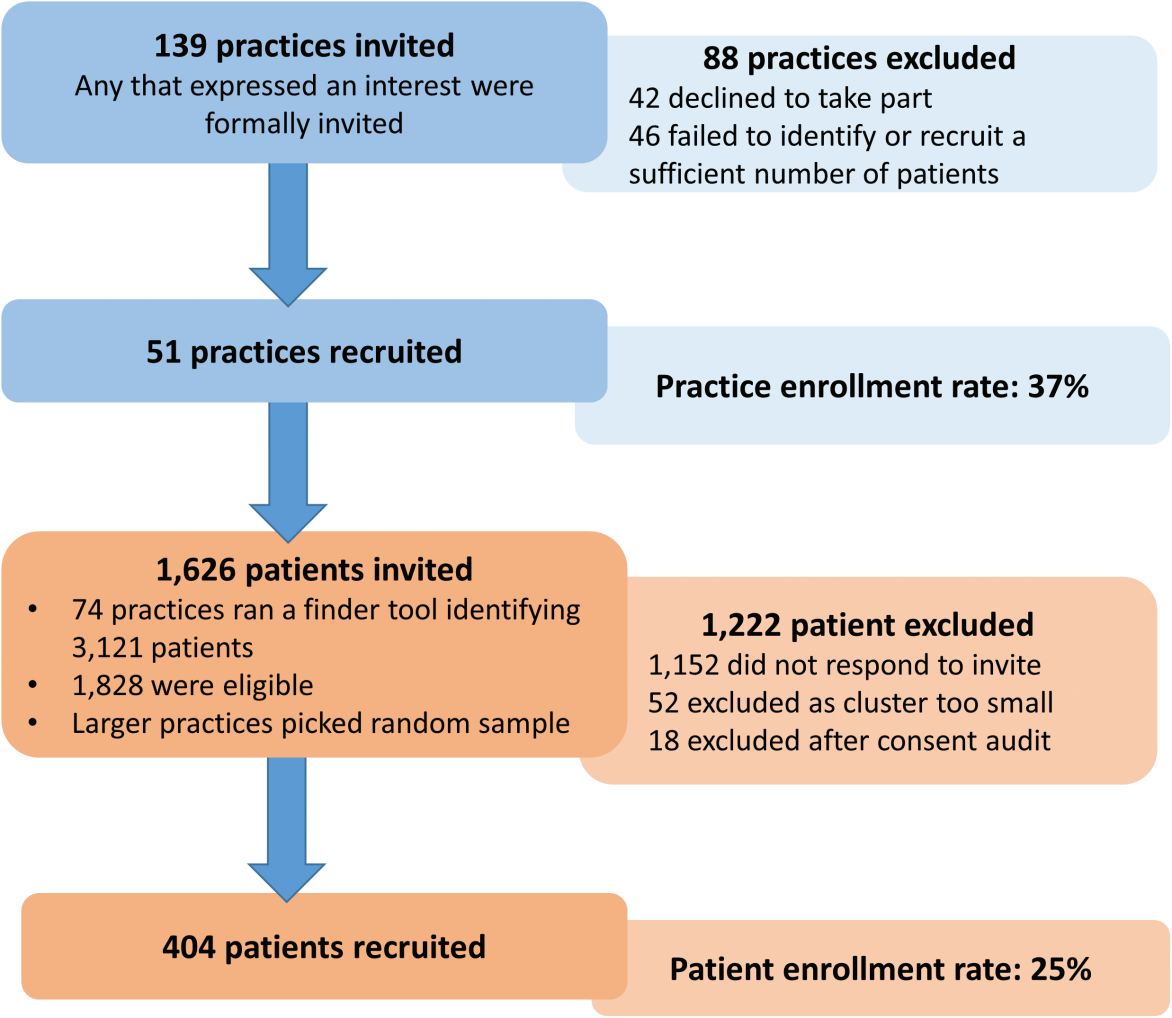
Practice and patient recruitment.

The present study includes data from 83 practices (all recruited practices [*n* = 51] and practices [*n* = 32]) that were recruited but failed to identify or enrol the required number of patients [Fig. 1]). At least 1 GP for each recruited intervention practice (*n* = 26), as well as a purposive sample of intervention patients (*n* = 46) were invited by telephone call, to participate in the process evaluation interviews. Eighteen GPs (69%) consented, 8 declined citing time constraint. Twenty-seven patients (58%) consented to participate in an interview, 11 declined citing fatigue or hearing difficulties and 8 did not reply.

### Data collection

Quantitative data obtained for the RCT included in this study were:

- Practice demographics collected from a practice profile questionnaire.
- Patient demographics collected from a postal questionnaire at study baseline.
- The number of invited practices and patients was collected by the study manager in line with CONSORT requirements.^22^
- Retention rates for recruited GPs (measured as completion rates for data collection of follow-up prescriptions).
- Retention rates for recruited patients (measured as completion rates for data collection of follow-up patient-reported outcomes).

Qualitative data were collected from semi-structured telephone interviews and study manager logs of GP or patient contact during the recruitment process. The study manager logged recurring themes from patients and practices, and individual contacts where the patient gave their study ID number. Motivations for and reservations about taking part were included in interview topic guides (see Appendices 1 and 2). Interviews were conducted by 2 female researchers who are also GPs (CMC, BK), and were trained by a senior researcher with qualitative experience (BC). Interviews were audiotaped and transcribed verbatim. Patient interviews lasted an average of 11.0 min and GP interviews lasted an average of 19.5 min. Participant data were anonymized, third-party references redacted and transcripts were coded with the practice or participant trial ID.

### Data analysis

Quantitative data were analysed using descriptive statistics in Stata (version 17, Stata Corp, College Station, TX). Practice and patient characteristics were compared with national sources to assess representativeness. Patient characteristics were compared with all over 65s in the 2016 national population census.^23^ Practice characteristics were compared with the most recently available data on the structure of general practices in Ireland.^24^ The Mann–Whitney *U*/Chi-squared tests were used to compare whether there were significant differences in practice characteristics between included and excluded practices.

Qualitative data were analysed thematically using NVivo 12. Transcripts were read repeatedly in order to achieve data familiarization, followed by line-by-line coding. The initial codes were developed by a researcher who was independent of the main trial (IP) and reviewed by CMC who was also the study manager. An inductive approach was employed through which codes were reviewed to assess commonality and differences across the data. As a result of the comparative analysis between and within transcripts, codes were labelled and organized into major subcategories and categories, which were synthesized into the main themes. The final categories and themes were reviewed, discussed and agreed upon by all members of the process evaluation study team.

## Results

### Recruitment rates

Recruitment ran over a 34-month period from March 2017 until November 2019, significantly longer than the 8 months originally anticipated. In total, 139 eligible practices were invited, 97 responded and ran the finder tool to identify eligible patients (14 did this before enrolment and were subsequently excluded as they failed to identify a sufficient number). Of the remaining 83 practices, 74 identified a sufficient number and posted patient invitations, 72 practices recruited at least 1 participant and 51 (enrolment rate 37%) recruited the required number of patients (Fig. 1). The median number of days from inviting patients to practice allocation was 142 (IQR 80.5–217.5). A total of 1,626 patients were invited to participate and approximately 70% (*n* = 1,152) did not respond. Four hundred and seventy-four patients were initially enrolled but 52 were excluded from 21 different practices that were unable to recruit at least 4 other participants and 18 patients were excluded after an audit of the consent process by the trial’s independent steering committee in light of new data protection regulations. Overall 404 patients were successfully enrolled (enrolment rate 25%).

Of the 26 intervention GPs, 18 were interviewed (11 of whom were male) and 8 failed to respond to interview invitations. Twenty-seven out of 208 intervention patients were interviewed, 12 of whom were male. The mean age was 73.7 years (SD 5.4), slightly younger than the mean for the entire group 76.5 years.

### Characteristics of recruited practices and patients

Compared with national practices, recruited practices were larger and more likely to be urban (Table 1). Practices that successfully recruited patients were larger, more likely to be urban, and more likely to have tighter GP involvement in the repeat prescribing process compared with practices that were excluded for not recruiting sufficient numbers of participants (Table 1). Comparing demographic details of participating patients to information on people aged ≥65 years from the 2016 population census,^23^ a larger proportion of trial participants were female and older and a smaller proportion were married. A higher proportion of participants had means tested eligibility for free primary medical care (Table 1).

**Table 1.** Comparison of included practices with excluded practices and national practices and comparison of recruited participants to all over 65s from the 2016 Irish population census.

Characteristic	Included participants	Comparison	*P* ^c^
	Recruited practices (*n* = 51)	National practices^24^ (*n* = 462)	
Five or more GPs in the practice, *N* (%)	20 (39)	88 (39)	0.001
Urban location, *N* (%)	30 (59)	194 (42)	0.021

	Recruited practices (*n* = 51)	Excluded practices^a^ (*n* = 32)	
Number of GPs, median (IQR)	4 (3–6)	3 (2–4.5)	0.027
Total number of patients, median (IQR)	6,420.5 (3,404–8,500)	4,500 (3,000–7,004)	0.074
Identified patients, median (IQR)	40 (24–55)	16.5 (10–25)	<0.001
Repeat prescribing policy, *N* (%)	25 (49)	14 (44)	0.640
GP generates prescription, *N* (%)	22 (43)	6 (19)	0.022
Urban location, *N* (%)	30 (59)	11 (34)	0.030
Practice manager, *N* (%)	42 (82)	25 (78)	0.635

	Recruited patients (*n* = 404)	2016 population census (*n* = 637,567)^d^	
Female, *N* (%)	230 (57)	340,960 (53)	0.160
≥80 years, *N* (%)	139 (34)	148,538 (23)	<0.001
GMS card, 65–69 years, *N* (%)	59 (83)	96,420 (46)	<0.001
GMS card^b^, ≥70 years, *N* (%)	272 (82)	333,551 (72)	0.128

IQR, interquartile range.

^a^These practices identified eligible patients and some were excluded at that stage as they did not identify enough eligible patients. Others started recruitment but were later excluded as they did not recruit enough patients.

^b^A general medical services (GMS) card is means tested and entitles the recipient to free GP visits and prescription medicines (aside from a €1.50 prescription charge). The income threshold for eligibility increases with age.

^c^Mann–Whitney *U*/Chi-squared tests.

^d^National Census 2016 Summary Results—Part 1, 2017.

Patient enrolment rates across the 51 practices varied considerably (median 35% IQR 24%–50%) (Supplementary Fig. 2). Urban practices had slightly lower enrolment rates but there was no significant difference in patient enrolment rates based on practice size or having a practice manager (Supplementary Table 1).

### Factors influencing GP recruitment and retention

#### Motivation to participate

GPs described a sense of agreement with the importance of the research question, “*the principle of what it was trying to achieve was something that I’m quite interested in anyway, which is polypharmacy, and you know really, trying to reduce the medicines burden on older people.*” (GP4) There was also concern for patient safety, particularly with respect to preventing adverse drug events and the challenge of co-ordinating care when multiple prescribers are involved, “*they’re attending various specialists in the hospital and they are just focusing on their area of specialty…..there are potential interactions. I suppose, maybe we’re the only central person to their prescriptions.*” (GP12) Some GPs were also motivated by the cost saving implications on a population level, “*you know saving expense to the health budget*.” (GP128) Others described feeling daunted by these prescriptions and saw SPPiRE as a way of addressing this, “*these very large prescriptions are very intimidating as well*.” (GP137) Participation in the SPPiRE intervention was seen as an opportunity to tackle inappropriate polypharmacy. GPs felt that their participation would potentially “*shine a light … and maybe pick up on something that we were doing wrong.*” (GP21)

#### Barriers to participation and recruitment of patients

The predominant reason reported for the 42 practices who declined to take part was insufficient time. While excluded practices could not be included in qualitative interviews, recruited GPs reported that potential barriers included lack of time and resources: “*The main thing is always the case with a slightly research type thing is trying to find the time to do it*.” (GP58) GPs described the recruitment process as time consuming and the complexity of the patient group added to this, “*we did try ringing a few patients but a lot of them they didn’t know why I was ringing and then when they had me on the phone, they didn’t want to talk about that, they actually wanted to discuss something else.*” (GP81) Only 6 GPs described involvement from other GPs within the practice, “*with the best will in the world it just didn’t get communicated to everybody*.” (GP39) The study manager logged 4 practices as having difficulty with patient identification, where prescription data had been input as free text in practice software and the finder tool did not function as a result. Practices also had difficulties identifying the total practice number of patients due to the lack of universal registration in Irish primary care. All randomized practices were retained, and barriers to retention postrandomization were not identified. However, intervention delivery varied and 3 practices failed to complete any of the medication reviews with their recruited participants, citing time constraints and lack of staff as the reasons.^19,21^

### Factors influencing patient recruitment and retention

#### Motivation to participate

Both interviewed GPs and patients indicated that direct contact from, and trust in their GP were the primary facilitators of patient recruitment. GPs felt that patients appreciated being contacted personally, “*I suppose my thinking on that was if the communication was coming from me that perhaps patients be more likely to agree to take part*.” (GP12) Some GPs mentioned that these were patients they were seeing regularly anyway and this facilitated recruitment. The importance of the relationship with the GP was also highlighted by patients, describing their GP as a figure who, “*personally care(s) about my health*” (GP4P13), “*would try to help me straight away*” (GP61P8), and whose advice would be followed unquestionably, “*I just go by whatever the doctor says*” (GP39P34). Trust and satisfaction with the relationship with a GP were echoed by 21 of the 27 patients. Similar to GPs, interviewed patients felt the research was important and taking part might be to their benefit, “*There might be something there that might you know help me out a bit, you know.*” (GP26P8)

#### Barriers to participation

The majority of patients who did not participate failed to respond to postal invitations. As these patients did not consent to participate, there is no demographic details to compare them to recruited participants. A barrier identified in study manager logs was concern about medicines being taken away. This concern was echoed by interviewed participants, though it did not prevent them from participating. Several participants had recently had lidocaine patches discontinued following national guidance that they be removed from state funded medicines schemes,^25^ and they were upset about this. Others highlighted that they were satisfied with their medicines and were afraid of any potential changes, “*It’s funny when you’re taking tablets and if it’s working for you….if it works don’t break it*.” (GP50P1) Study personnel contacted potential participants who had questions about the trial with their consent, and that of their GP. In keeping with the theme of trust identified as a facilitator, some patients (that were subsequently recruited) expressed concern that the purpose of the study was to monitor their GPs’ performance and they were not comfortable with that. Study manager logs recorded contact from 3 GPs who felt their patients would not have the literacy skills to understand information leaflets. This was corroborated by the experience of the study manager when discussing the trial with interested patients and when reviewing completed trial documentation. Difficulty with filling in documentation was also outlined by 1 patient, “*The one thing I hate doing is filling out forms….when I go to a place and there is a big form, I always ask them for help …*” (GP46P5).

#### Barriers to retention

Thirty-five patients (8.6%) were lost to follow-up for primary outcomes, mainly because they had died and 45% for patient-reported outcome measures (PROMs) as they failed to respond to follow-up postal questionnaires (Table 2). Interviewed participants described significant disease and treatment burden, “[I] *spend 15 minutes or 20 minutes or whatever it is every morning, making sure that I take the right tablets*.” (GP39P34) GPs identified this as a barrier to both recruitment and retention, “*I think there were one or two that might have dropped out but it was because they were sick and they weren’t able for it.*” (GP61)

**Table 2.** Summary of GP and patient motivations and barriers to recruitment and retention.

Subtheme	Data source and examples where relevant
GP motivation: perceived importance of the research question	
Concern for patient safety	GP interviews “*we’ve had a few episodes of adverse drug reactions in the practice with (the) elderly as well, so I suppose that’s what got me motivated to get involved*” (GP12)
Reduce treatment burden	GP interviews “*And it’s such a burden on people the medications and they become so used to it and, you know, they go into hospital and come out on another three different tablets and they’re into a clinic a different consultant puts them on something else, so just a really good option to cut down on that burden for a lot of patients*.” (GP61)
Re-evaluate current practices	GP interviews “*I suppose we felt it probably will increase our own awareness of polypharmacy and drug interactions and maybe just make us more aware of doing repeat prescriptions whether we really needed to be prescribing everything we were prescribing.*” (GP26)
GP barriers	
Lack of time and resource	Study manager logs, GP interviews “*So it is the usual in GP, time and resources is the biggest issue*.” (GP81) “*I was wary maybe about the workload involved and maybe the amount of time that I might have to give over towards it*.” (GP21) “*The downsides, well obviously the resources, the time resources involved.*” (GP122)
Recruitment processes	Study manager logs, GP interviews • Finder tool did not function as anticipated in at least 3 practices • Little involvement or adaptation of the rest of the practice “*So we had to identify patients that was the first thing. Um, we then had to sort of try to kind of send the invitations out, contact the patients. Chase them up a little bit, accept that there they weren’t all going to turn up for them in terms of doing it as part and parcel of the project. Look at those who were unsuitable. So even before you started there’s a certain amount of time involved*.” (GP58)
Complexity of the patients	“*I definitely think they found a little bit harder to get into me then when they’re in, they’re kind of like, well, I’m here now I want my full, OT, I want my bloods and everything. And you’re trying to explain, you know, that no that’s not why you were asked to come in today. But then when people are doing you a favour …*” (GP81)
Patient motivations	
Direct contact from the GP	GP interviews “*I practice in a rural part of the country and in some respects when the GP phones these people up they are very accepting of something you’re trying to do. So, there was good cooperation initially … they were good when they came in and they were very appreciative of what we were trying to do*.” (GP21)
Trust in the GP	GP and patient interviews “*I think patients that you see regularly and most of these patients that are, you know on polypharmacy you would have built up a relationship with them over the years because they would be people that you would be seeing quite often*.” (GP26) “*I let him be the boss. He’s the man that should know you know what I mean like*.” (GP26P8)
Potential benefit from participation	Study manager log, patient interviews “*Because I thought I was getting too much tablets like really, you know*.” (GP26P8) “*I’m interested in taking part because I am on a lot of medication … people sometimes say to me ‘are you drunk’ because I was staggering from side to side … (male patient from GP92)*” (study manager log, August 2018) “*Because I would have an interest in medicine and worked in a hospital environment in my younger days and always found the subject interesting and I feel that any research that can be part of, I’ve always been willing to participate in it*.” (GP39P41)
Patient barriers	
Concern about medicines being taken away	Study manager logs, patient interviews “…*if the HSE are doing it to save money and want to take people off medication they’re on, then that’s a different kettle of fish*.” (GP4P13) “*All I was afraid about of was … one doctor one consultant at one stage did take me off a lot of my medications. They said I was on too much. But within a short period of time I found out I was back on all of it again. I would be concerned that I would be taken off something that would be vital*.” (GP39P41) “*GP34 reported that many of his patients were concerned their medicines would be stopped by the HSE (in light of recent media coverage around Versatis patches).*” (study manager log, 09/03/2018)
Difficulty with trial documentation	Patient interview, study manager logs “*GP85 and 121 feel there is poor health literacy and many put off by questionnaire and leaflet*.” (study manager log, October 2018)
Complexity of patients	GP and patient interviews, study manger logs “*We had to put in quite a lot of work to make sure that they would attend. So they continuously forgot, or the relatives couldn’t bring them*.” (GP97) “*Anger over difficulty to get home help and fear of losing home help. ‘How do you propose I keep my house clean?’ (Woman with home help 3 times per week, very poor mobility).*” (study manager log) “*Constantly running to the loo especially early in the day not being able to leave the house. It can be a bit of a nuisance. More than a nuisance. Now, trying to time things for the evening and appointments and stuff like that. And I drive, so I’m in the process of having cataracts done at the moment. Not being able to drive because I can’t use public transport, because when my back gives I just have to sit down*.” (GP39P41)
Trust in the GP and suspicion of external influence	Study manger logs from contact with patients who had questions about participation and their GP directed them to study personnel “*GP8’s patients reported concern that we were monitoring his prescribing. Many made a point of reassuring me he was a very good GP*.” (study manager log, 09/03/2018)

## Discussion

### Summary of the results

**Fig. 2.**
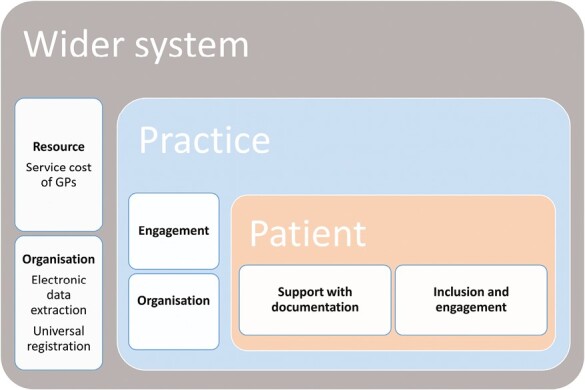
Summary of factors influencing GP and patient recruitment.

Despite reaching recruitment targets, there were challenges and delays in the recruitment process and 45% of patients were lost to follow-up for PROMs. Forty-six practices were excluded before allocation because they failed to identify or recruit a sufficient number of patients. Quantitative data indicated that practices that successfully recruited had more GPs and were more likely to be urban. Recruited practices were more likely to have a policy that involved more direct GP engagement in the repeat prescribing process, indicating these practices were more likely to adhere to national guidance around repeat prescribing.^26^ GPs perceived the research question to be important and this was their primary motivation for participation. The predominant barrier for GP recruitment was lack of time and this was compounded by the complexity of the patients and the organizational infrastructure of Irish primary care. Patients were influenced to participate based on a trusting relationship with their GP, although many reported that they also felt the research question was important and they may benefit from participation. Wider practice engagement with the recruitment process, for example assisting with trial documentation facilitated patient recruitment. The patient enrolment rate was 25%, the majority of patients who did not take part failed to respond to postal invitations. Potential barriers to patient recruitment included difficulty with trial documentation, treatment and disease burden, suspicion of external influence and fear of medicines being taken away. The latter barrier was specific to the SPPiRE intervention which had a depresciribng approach, however fear of missing out on effective therapies if allocated to the control group has been identified as a barrier to participation in other studies.^27^ In addition to literacy difficulties comorbid conditions such as visual impairment, osteoarthritis of the hands, and early memory and cognitive difficulties may have further affected the ability to complete postal questionnaires. Progression of these difficulties over the study period may explain the large loss to follow-up of PROM data compared with baseline. Figure 2 summarizes the relationship between the wider system, the practices and patients on recruitment.

### Comparison with existing literature

The enrolment rate of GPs was high compared with other national studies,^28^ however SPPiRE GPs had expressed an interest in participating before being formally invited so it is difficult to draw conclusions from the comparison. Similar to SPPiRE, qualitative work with GPs in an Irish research and teaching network identified perceived relevance and importance of the research question as important motivators for GP participation in research and lack of time and resource as barriers.^29^ Another qualitative study in the Netherlands designed to identify the facilitators and barriers of patient recruitment as experienced by GPs, concluded that improvements could be made by ensuring study processes are simple and require limited time investment from the GP.^17^ This needs to be balanced with ethical, legal, and regulatory requirements. Three cluster RCTs based in other primary care settings described difficulty with patient recruitment where GP time and prioritization of recruitment were identified as major causes.^16,30,31^ A UK survey of 572 GPs indicated that organizational infrastructure and working environment were important factors in encouraging GP participation in research.^32^ Primary care research output in Ireland has lagged behind other European countries and not meeting the service costs of GPs participating in research has been identified as a reason.^33^ Experiences from SPPiRE corroborate these findings that significant time, resource, and planning are needed for successful patient recruitment to primary care RCTs.

Many patients described complete trust in their doctors and decided to take part simply because their GP had asked. Trust (along with altruism and personal benefit) has been identified as one of the 3 dominant facilitators of patient recruitment which is relevant across many settings and study designs.^27^ Interestingly this aspect of the doctor–patient relationship has been identified as a barrier to shared decision-making,^34^ and this was identified as a theme when analysing how SPPiRE was implemented and exerted its effect, with many GPs and patients not engaging in the discussion around the treatment prioritization component of the intervention.^21^ Trust in the opinions of care givers and physicians has also been identified as a barrier to participation where the advice from the trusted source is not to participate.^15^ Difficulty understanding trial documentation was identified as a barrier to patient recruitment in SPPiRE. Other studies have identified that participants’ comprehension of consent components is low.^35^ This raises ethical concerns for research in vulnerable populations, and emphasize the importance of allocating resources to support recruitment, particularly for higher-risk interventions. Barriers to retention of older patients in RCTs identified in SPPiRE and other studies include disease and treatment burden which are often compounded by long study duration and difficulty accessing the study site.^36^

A qualitative evidence synthesis of pretrial research concluded that there needs to be a move beyond describing the problems with recruitment and retention, to suggesting improvements.^37^ Strategies include ensuring patient and public involvement in both the development and conduct of clinical trials,^38^ the use of incentives, awards, and motivational information,^39^ acknowledging extended time frames and planning for higher resourcing costs.^40,41^ Older patients with multimorbidity are the highest consumers of medical interventions but are under-represented in experimental research.^42^ Experiences from SPPiRE highlight that recruiting and retaining this population through primary care is possible but requires significant recourses and planning and particular attention to the consent process and trial documentation.

### Strengths and limitations

This study includes quantitative data and patient, GP and study personnel perspectives on recruitment and gives an insight into the particular difficulties faced by pragmatic cluster RCTs involving patients with significant disease and treatment burden.

The primary aim of the trial’s process evaluation was to examine intervention implementation, so only intervention GPs and patients were invited to participate in interviews. These interviews took place after the intervention period, on average at least 6 months from recruitment, meaning some participants may have had difficulty recalling the recruitment period. The process evaluation was carried out parallel to the main trial, to reduce the likelihood investigators may be more focussed on explaining the results rather than unearthing unintended consequences. As a result interviewed participants had not yet returned follow-up questionnaires and so the reasons for the low response rate at follow-up was not explored in the interviews. While excluded practices and patients views could not be included in this analysis, themes from the interviews corroborate the researcher logs. One of the interviewers (CMC) was also the study manager and had contact with intervention GPs during the RCT, increasing the risk of social desirability bias.

### Implications for future research

The GP enrolment rate in this study was high, and GPs identified perceived importance of the research question as the main motivation for participation. Including the clinician’s perspective at all stages of the research process from conceptualization and funding applications to study design and dissemination is important, particularly when they are the direct target of the intervention. Collaborative models that incorporate the perspective of full-time clinicians such as the newly formed “Primary care Academic CollaboraTive” (PACT) in the United Kingdom may help facilitate this.^43^ Despite high enrolment rates and interest in the study many GPs failed to recruit sufficient number of patients. We only had resources to support practice recruitment remotely. Supporting practices in terms of resource and organization is important to facilitate recruitment, particularly giving additional time and support for recruitment of hard to reach populations. This is especially important in primary care where there is a real opportunity to include hard to reach groups. Similar to GPs, patients were motivated to participate as they felt the research was important and there was a chance they may directly benefit. Meaningful patient involvement in research priority setting, and study design is important, facilitates involvement, ensures research questions are relevant and has rightly become routine in many settings.^44^ Finally, we identified the administrative work involved in participation as a barrier to patient recruitment, and this is especially the case for vulnerable groups who often have higher levels of multimorbidity and lower literacy levels. Futures studies involving these groups need to pay careful attention to trial documentation and resource clinicians involved in recruitment so they can give the time and attention needed to support potential patient participants.

## Conclusion

The SPPiRE trial successfully recruited older patients with significant disease and treatment burden. Contact from their GP was a major facilitator of patient recruitment indicating that primary care is the ideal environment for clinical research in hard to reach groups. The recruitment period was significantly prolonged and GPs identified lack of time and resource as major barriers. Adequate planning and resource allocation are needed to support GPs in recruitment of vulnerable patients participating in primary care research, which is critical to generating evidence that can inform real-world clinical practice.

## Supplementary Material

cmad039_suppl_Supplementary_Material

## Data Availability

The data underlying this article will be shared on reasonable request to the corresponding author.
