# Supplementary material for: Recruiting general practitioners and older patients with multimorbidity to randomized trials
Source: Fam Pract. 2023 Apr 4;40(5-6):810–9. doi: 10.1093/fampra/cmad039 (PMC10745264; doi:10.1093/fampra/cmad039)
Supplement: cmad039_suppl_Supplementary_Material [file cmad039_suppl_supplementary_material.pdf]

Supplementary material

Supplementary Figure 1, overview of the evaluation of the SPPiRE intervention

Effectiveness evaluation

Design and setting
Cluster RCT based in Irish primary care
Population
General practices, nationwide that had at least 300 older people on their patient panel
Patients aged ≥65 years and prescribed ≥15 medicines
Intervention group (26 practices, 208 patients)
Online professional training videos
GP delivered, web guided medication review
Control group (25 practices, 196 patients)
Usual GP care, at the time there was no structured chronic disease management programmes in Irish primary care
Outcomes
Small but significant reduction in the number of medicines (IRR 0.95, 95% CI: 0.899 to 0.999, p = 0.045) but no significant effect on the odds of having at least 1 PIP (OR 0.39, 95% CI: 0.140 to 1.064, p = 0.066)
No harms associated with the intervention

Parallel mixed methods process evaluation

Cost effectiveness evaluation

Appendix 1

SPPiRE Interview Topic Guide – Intervention GPs

Prescribing for older patients

- Can you talk me through how repeat prescriptions for older patients are reviewed in your practice?
    - Dedicated medication review visit Vs opportunistic
    - Who does it?
    - How are changes made when reviewing repeat prescriptions?
    - If problems are identified how are they managed, eg message left for patient/pharmacist through admin staff or GP contacting patient or written message left for patient with prescription?
- Can you describe some of the issues you face when reviewing prescriptions for these patient?
    - Uncertainty over who started the medicine and why
    - Uncertainty over potential benefits and risks
    - Reluctance to make too many changes due to fear of adverse consequences/the need for added visits
    - Patient preferences/demands
    - Previous positive or negative experiences of changing or discontinuing repeat medicines

Summary of intervention use

**NPT Construct**	**Questions/Prompts**
**Coherence:** making sense of the intervention	What did you think would be the benefits of taking part in SPPiRE?<br>What were you wary of in terms of taking part?<br>How did you see these medication reviews fitting into your day to day practice?
**Cognitive Participation:** involvement with the intervention	Can you describe who else was from the practice was involved in the SPPiRE intervention. What do you feel their views of this were?<br>What did you see your role as being?<br>Describe how the practice has had to adapt to incorporate the implementation of the intervention.<br>Can you tell me what you thought about the training videos?
**Collective Action:** how practical work of *doing* the intervention is carried out within the organization	Can you talk me through the steps that were involved in performing the medication review?<br>• How were the appointments arranged, did the patients turn up?<br>• Use of the SPPiRE website?

	• Was the review performed in one sitting? Was the patient present?<br>• What worked well? What didn't work so well? Was the website easy to use?<br>• PIP identified? How did patient respond to suggested changes?<br>• Brown bag<br>• Patient priorities<br><br>Can you describe how compatible this was with existing practice?
**Reflexive Monitoring:** evaluation and appraisal of the intervention	Overall how would you describe your experience of taking part? Was it worthwhile? Would you like to see this or something similar in routine use (if yes, any suggestions as to what changes would be needed to improve intervention, if no reasons why it would be unfeasible in routine practice)?<br><br>What you would change?<br><br>In what way do you think it has had any impact?<br><br>Are there any aspects of the intervention that you have now incorporated into your routine practice?<br><br>Added workload, ADWEs?

Concluding comments

- Overall is there anything else you would like to comment on?

Appendix 2

SPPiRE Interview Topic Guide – Intervention Patients

Medications in general

- You were invited to take part in this study because of the number of medicines you are currently prescribed. How do you feel about the number of medicines you are prescribed?
    - What do you think is a lot of medicines to take?
    - How important/necessary do you feel they are?
    - Do you feel you know what medicines you are taking and why?
    - If you had a concern about your medicines, who would you talk to?
    - Do you feel you can talk to your GP about your medicines?
    - Do you like to be involved in decisions about your medicines?

Summary of intervention use

- As part of this study you would have been invited to attend a medication review visit with your GP.
    - What did you expect would happen?
    - Can you describe how that visit went?
    - Did you bring your medicines in with you to the visit?
    - How were your ideas, concerns and priorities addressed?
    - Were any changes made to your medicines? How did you feel about this?
    - Which things did you like the best about it? What did you not like/what would you change?
- What, if any, difference do you feel it has made to you
    - happier about medicines
    - reassured they were reviewed
    - concerns and priorities were addressed
    - left you feeling concerned or worried about your medicines
- Would you like to see something like this routinely used?
    - If yes, any suggestions are to how to improve or sustain it?
    - If no, why?

Concluding comments

Is there anything else the participant would like to add?

Supplementary Figure 2, patient enrolment rates by practice

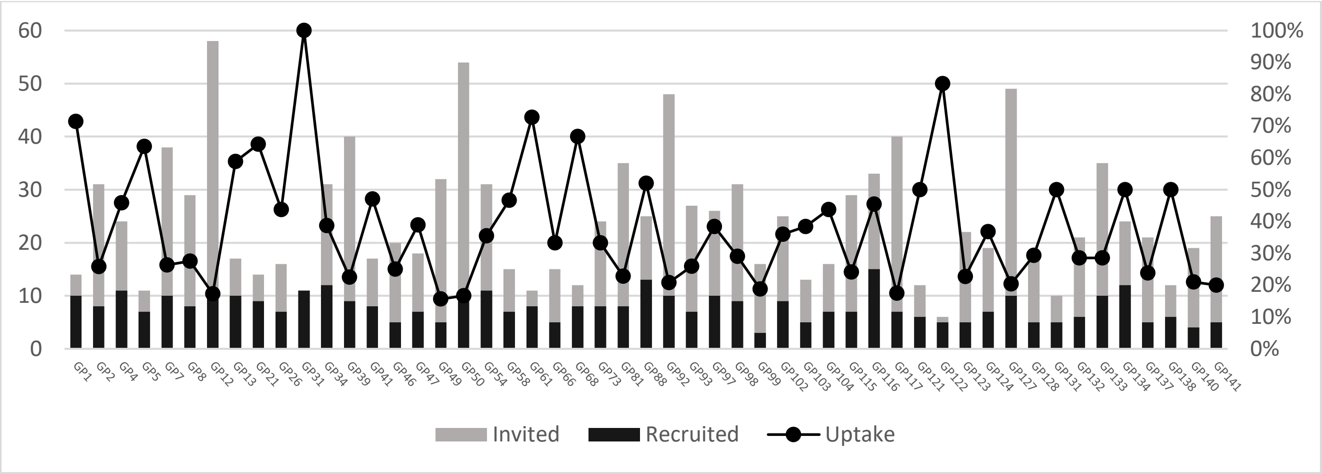

Supplementary Table 1, patient enrolment rates by practice characteristics

Percentage of invited participants recruited		Mean difference (95%, CI)
Urban (n=30)	Non-urban (n=21)	
33.8%	45.3%	11.5% (1.2%, 21.8%)
> 30 GP sessions per week (n=20)	< 30 GP session per week (n=31)	
39.9%	37.6%	-2.3% (-13.1%, 8.6%)
Practice manager (n=42)	No practice manager (n=9)	
39.8%	32.8%	-7.0% (-20.8%, 6.8%)

Consolidated criteria for reporting qualitative studies (COREQ): 32-item checklist

Developed from:

Tong A, Sainsbury P, Craig J. Consolidated criteria for reporting qualitative research (COREQ): a 32-item checklist for interviews and focus groups. *International Journal for Quality in Health Care*. 2007. Volume 19, Number 6: pp. 349 – 357

**No. Item**	**Guide questions/description**	**Reported on Page #**
Domain 1: Research team and reflexivity		
Personal Characteristics		
1. Interviewer/facilitator	Which author/s conducted the interview or focus group?	Page 7, (Methods, Data collection)
2. Credentials	What were the researcher's credentials? E.g. PhD, MD	Page 7, (Methods, Data collection)
3. Occupation	What was their occupation at the time of the study?	Page 7, (Methods, Data collection)
4. Gender	Was the researcher male or female?	Page 7, (Methods, Data collection)
5. Experience and training	What experience or training did the researcher have?	Page 7, (Methods, Data collection)
Relationship with participants		
6. Relationship established	Was a relationship established prior to study commencement?	The study manager had contact with GPs during the intervention period but had little contact with patients, unless necessary to collect missing data or review the consent process.<br>Page 15, (Discussion, Strengths and limitations)
7. Participant knowledge of the interviewer	What did the participants know about the researcher? e.g. personal goals, reasons for doing the research	Not recorded.<br>Participants were given information about the names, professional background and

		credentials of the research team in information leaflets which they read prior to consenting to take part.
8. Interviewer characteristics	What characteristics were reported about the interviewer/facilitator? e.g. Bias, assumptions, reasons and interests in the research topic	Both interviewers were GPs, one was also the study manager.<br>Page 7, (Methods, Data collection)

Domain 2: study design		
Theoretical framework		
9. Methodological orientation and Theory	What methodological orientation was stated to underpin the study? e.g. grounded theory, discourse analysis, ethnography, phenomenology, content analysis	Page 7, (Methods, Data analysis)
Participant selection		
10. Sampling	How were participants selected? e.g. purposive, convenience, consecutive, snowball	Page 6 (Methods, Study Population)
11. Method of approach	How were participants approached? e.g. face-to-face, telephone, mail, email	Page 6 (Methods, Study Population)
12. Sample size	How many participants were in the study?	Page 6 (Methods, Study Population)
13. Non-participation	How many people refused to participate or dropped out? Reasons?	Page 6 (Methods, Study Population)
Setting		
14. Setting of data collection	Where was the data collected? e.g. home, clinic, workplace	Page 7 (Methods, Data Collection)<br>GPs were telephoned at their work-place and patients at their home.
15. Presence of non-participants	Was anyone else present besides the participants and researchers?	No

16. Description of sample	What are the important characteristics of the sample? e.g. demographic data, date	Page 8-9, (Results, Characteristics of recruited practices and patients)
Data collection		
17. Interview guide	Were questions, prompts, guides provided by the authors? Was it pilot tested?	Appendix 1 and 2
18. Repeat interviews	Were repeat interviews carried out? If yes, how many?	No
19. Audio/visual recording	Did the research use audio or visual recording to collect the data?	Yes, interviews were recorded.<br>Page 7 (Methods, Data Collection)
20. Field notes	Were field notes made during and/or after the interview or focus group?	No
21. Duration	What was the duration of the interviews or focus group?	Page 7 (Methods, Data Collection)
22. Data saturation	Was data saturation discussed?	No
23. Transcripts returned	Were transcripts returned to participants for comment and/or correction?	No. Participants were offered a copy of the transcript but none requested it.
Domain 3: analysis and findings		
Data analysis		
24. Number of data coders	How many data coders coded the data?	Two<br>Page 7, 8 (Methods, Data Analysis)
25. Description of the coding tree	Did authors provide a description of the coding tree?	No
26. Derivation of themes	Were themes identified in advance or derived from the data?	Derived from the data.<br>Page 7- 8 (Methods, Data Analysis)
27. Software	What software, if applicable, was used to manage the data?	Stata Version 17 and NVIVO 12

		Page 7 (Methods, Data analysis)
28. Participant checking	Did participants provide feedback on the findings?	No
Reporting		
29. Quotations presented	Were participant quotations presented to illustrate the themes/findings? Was each quotation identified? e.g. participant number	Yes, Pages 9-12 (Results)
30. Data and findings consistent	Was there consistency between the data presented and the findings?	Yes, Pages 9-12 (Results)
31. Clarity of major themes	Were major themes clearly presented in the findings?	Yes
32. Clarity of minor themes	Is there a description of diverse cases or discussion of minor themes?	Yes
